# Developing harm reduction in the context of youth substance use: insights from a multi-site qualitative analysis of young people’s harm minimization strategies

**DOI:** 10.1186/s12954-017-0180-z

**Published:** 2017-07-31

**Authors:** Emily K. Jenkins, Allie Slemon, Rebecca J. Haines-Saah

**Affiliations:** 1 School of Nursing, University of British Columbia, T201-2211 Wesbrook Mall, Vancouver, British Columbia V6T 2B5 Canada; 2 Community Health Sciences, University of Calgary, 2500 University Drive NW, Calgary, Alberta T2N 1N4 Canada

**Keywords:** Youth, Substance use, Harm reduction, Harm minimization, Multi-site qualitative research, Social context, Drug prevention

## Abstract

**Background:**

Youth substance use programming and educational strategies are frequently informed by prevention approaches that emphasize abstinence goals, which often do not resonate with youth in their lack of acknowledgment of young people’s social context and how young people perceive positive effects of substance use. Further, approaches to drug prevention have been critiqued as adopting a one-size-fits-all approach and therefore inadequate in addressing substance use in the context of population variation and inequities. In response to the limitations of current approaches to prevention, programming informed by harm reduction principles that aims to minimize harms without requiring abstinence is emergent in school settings. However, youth perspectives informing harm reduction are limited in both research and program development.

**Methods:**

This paper draws on data from the Researching Adolescent Distress and Resilience (RADAR) study, which utilized an ethnographic approach to bring youth voice to the literature on mental health and substance use. Qualitative data collection included individual interviews (*n* = 86) with young people aged 13–18 across three communities—representing urban, suburban, and rural geographies—in British Columbia, Canada. A multi-site qualitative analysis of interview data was conducted to identify themes across and within each research site.

**Results:**

Across all three sites, young people’s individual experiences of substance use were shaped by geographic, socio-cultural, and political contexts, with youth describing their use in relation to the nature of substance use in peer groups and in the broader community. To manage their own substance use and reduce related harms, youth employed a variety of ad hoc harm minimization strategies that were reflective of their respective contexts.

**Conclusions:**

The findings from this study suggest the importance of harm reduction approaches that are contextually relevant and responsive to the lived experiences of youth. Youth perspectives in the development of harm reduction programming are needed to ensure that approaches are relatable and meaningful to young people, and effective for promoting the minimization of substance-related harms.

## Background

Substance use, including both alcohol and drug use, is identified internationally as a key issue affecting youth populations. The World Health Organization (WHO) reports that heavy episodic drinking is higher among youth ages 15–19 than in the adult population (11.7 versus 7.5%) [1], and the United Nations [2] characterizes illicit drug use as a “youth phenomenon”, with rates of use increasing through teenage years to a peak in early adulthood. In Canada, prevalence of substance use echoes that of global studies: 40% of youth in grades 7 to 12 reported past-year alcohol use, 17% cannabis use, and 1–4% used other illegal drugs [3]. The potential health impacts of substance use among youth include injury or death [4], over-consumption [5], and consumption of drugs with unknown potency or contents, a growing concern within the context of a “contaminated” illicit drug supply [6, 7]. Early onset and frequency of substance use in adolescence has also been linked to risk for mental health challenges and increased problematic substance use in adulthood [8, 9].

The health risks and harms associated with youth substance use have prompted a proliferation of universal prevention programs in schools, which predominantly approach substance use through an abstinence and avoidance lens [10, 11]. Drug and alcohol prevention has also been utilized to target populations identified as at risk or marginalized (i.e., indicated prevention), including youth in the foster care system [12], LGBTQ youth [13], and youth in racialized communities [14, 15], for example. Some evidence has demonstrated that indicated prevention approaches applied to particular youth contexts may reduce frequency or amount of use [16, 17]; however, prevention programs have been critiqued for their narrow focus on preventing or avoiding use, as opposed to equipping youth with skills in identifying and mitigating harms that may occur within the context of choosing to use [18, 19]. Bok & Morales [20] argue that in social contexts in which drug use is positively reinforced by peers, and in which youth endorse positive effects of drug use, abstinence and prevention approaches to drug education are “a form of denial, by adults, about the actual behaviors of teenagers and young adults” (p. 94). Further, prevention programming frequently presents alcohol and drug information in a manner that may not resonate with the intended youth audience, for example highlighting potential negative consequences as a deterrent strategy or “scare tactic”, rather than acknowledging and addressing social contexts of use [21, 22]. Prevention approaches also fail to acknowledge that youth frequently use substances for pleasure and enjoyment of intoxication, instead framing substance use as a sign of distress or lack of common sense [23, 24]. Consequently, youth perspectives on current substance use prevention programs demonstrate a lack of trust in formal sources of alcohol and drug information, with higher trust in information from peers [22, 25]. Drug education programs such as D.A.R.E. (Drug Abuse Resistance Education), which are led by adult authority figures such as police officers and centre fear-based messaging, are widespread yet are unrelatable to young people in their mode of delivery and ineffective at minimizing substance use harms [26, 27].

Contemporary harm reduction emerged in the early 1990s, but is still a nascent, alternative orientation to youth substance use programming. This approach holds potential to address some of the shortfalls of existing prevention programs, although it remains contentious in the context of youth substance use and has not been widely studied within this population [17, 19]. Harm reduction includes abstinence goals, but is based on a philosophy of “starting where the user is at” and “offers a pragmatic yet compassionate set of strategies designed to reduce the harmful consequences” [28] (p. 779) of substance use for both the substance user and the broader community. Given this, it can be directed across the spectrum of substance use and may be applied at the individual, community, or societal level [29]. In addressing minimization of harms within the context of use, the philosophy of harm reduction differs from traditional approaches of prevention and abstinence, which have been largely oriented at reducing or preventing use altogether. Harm reduction approaches to youth substance use, while still limited in practice, have started to emerge in school settings in response to criticisms of prevention programs as ineffective for engaging youth in meaningful dialogues on substance use and for failing to acknowledge and tailor messaging and approaches to variations in youth contexts [26, 30]. Examples of recent harm reduction approaches in schools include SHAHRP in the UK [31], DEVS in Victoria, Australia [32], and SCIDUA in Eastern Canada [33], all of which aim to increase substance use knowledge and target the reduction of associated harms rather than frequency or amount of use directly. Each program has resulted in safer attitudes toward substance use, and reduced incidents of harms associated with alcohol and drug use, providing an evidence base for shifting the framework of school-based substance use education from prevention to harm reduction.

Outside of programming specifically directed to vulnerable youth and youth using injection drugs (e.g., [34]), youth perspectives on harm reduction are frequently absent from the conversation informing these approaches [21, 30]. With limited research centering youth experiences of and perspectives on drugs and alcohol, educational strategies for addressing the harms of youth substance use risk continuing with a top-down approach that is not informed by or relevant to youth context [22]. In this article, we take an initial step at addressing these important gaps, presenting findings from the Researching Adolescent Distress and Resilience (RADAR) study, a qualitative study involving youth across three communities in British Columbia (BC), Canada. By presenting a multi-site qualitative analysis of youth reflections on the social and community contexts for their experiences of substance use and on their own harm minimization strategies, this study brings youth perspectives to the harm reduction literature and speaks to the importance of contextually relevant harm reduction programming.

## Methods

The analysis reported in this paper draws on data from a qualitative study conducted in BC, Canada, from 2012 to 2014. The RADAR study used ethnographic approaches including participant observation and field notes and individual interviews to bring youth perspectives to the mental health and substance use literature to inform policies, practices, and programs targeting adolescents. Ethnographic approaches were selected because they support comprehensive understandings of participants’ beliefs and behaviours as well as an understanding of the context within which these were established [35]. Data analysed in this article were drawn from individual interviews that were conducted with 83 young people ages 13–18. Data collection occurred at three research sites across the province, representing urban, suburban, and rural geographies. The sites were selected to represent diversity in geography, community history, societal and cultural factors, and demographics. This paper presents a multi-site qualitative analysis of all three study sites, which are described in detail below.

### Study sites

The three research sites are identified by pseudonyms—The City, The Valley, and The North—to protect the confidentiality of study participants and members of their communities. The City is a large urban centre with a population of approximately 604,000 within a metropolitan area of just under 2.5 million [36]. The City is culturally and ethnically diverse: 48% of the population are first-generation immigrants, and over half are a visible minority including 27% from Chinese descent [37]. The City’s leading industries are tourism, trade, and film. The per capita alcohol consumption in The City was 8.60 l in 2015, though varies widely by neighbourhood (3.31–16.79); the particular neighbourhood from which the participant sample was drawn was close to The City mean [38]. Rates of drug use by region are not available in BC, but drug-related hospitalizations provide a measure of substance use-related harms and are reported at 40.2 per 100,000 in 2015 in the study neighbourhood (40.2–427.38 in The City) [39].

The Valley is a suburban city with a population of approximately 133,000, located proximally—within 75 km—to a large urban centre [36]. Seventy-three percent of The Valley’s population is religiously affiliated, with Christianity and Sikhism as the two most prominent religions—a contrast to The City, in which only 50% of the population identifies as religious [37]. The Valley’s ethnic composition is predominantly of European descent (64%), with a large South Asian community (22% of total population; 76% of visible minorities). Punjabi is spoken in one fifth of households [37]. Over one quarter of the population are first-generation immigrants. The main industry in The Valley is farming, with most of the municipality’s land zoned as agricultural. Per capita alcohol consumption in The Valley was 6.57 l in 2015 [38], and drug-related hospitalizations were 189.3 per 100,000 in this same year [39].

The population of The North is approximately 4800, including individuals living in town, on surrounding First Nations reserve land, and more rural areas [40]. In addition to the individuals living on reserve land, 27% of the population in town identifies as “Indigenous” or “First Nations”, in comparison to 2–3% in The City and The Valley [37]. The North’s main industries are forestry, mining, and agriculture. Per capita alcohol consumption in the local health area in which The North is situated was 9.86 l in 2015 [38], and drug-related hospitalizations were 175.6 per 100,000 [39].

### Data collection

Ethics approval for the study was obtained through the University of British Columbia, and permission was granted from the respective school districts in each community. Each study site had one affiliated high school and youth were recruited through flyers posted within the school and through the assistance of school and community staff. Recruitment efforts were directed at ensuring a diverse sample of participants with respect to age, gender, ethnicity, and experience with mental health or substance use issues. To protect privacy and support confidentiality, youth provided their own consent to participate and did not require parent/guardian permission. Individual interviews were conducted by members of the research team on-site at each school in a private space. Interview questions aimed to elicit participant accounts of emotional distress and resilience as well as the contextual factors shaping these experiences. Interviews began with a grand tour question with probes used to gain further depth and clarity as necessary. Interview length ranged from 30 to 120 min and participants were given $20 CAD honoraria to acknowledge their time and contributions. Interviews were subsequently transcribed, with pseudonyms used to protect the confidentiality of participants.

The demographics of study participants were as follows: In The City, 29 youth participated in the study: 14 females, 13 males, 1 “non-binary identifying”, and 1 who intentionally left the gender information blank; 16 participants identified as “White”/European, 5 multi-racial, 3 Asian, 1 Latin American, 1 West Asian, and 3 did not provide details. In The Valley, 27 youth participated: 13 females and 14 males; 12 participants identified as South Asian, 11 “White”/European, 1 “Black” (e.g., of African or Caribbean descent), 2 multi-racial, and 1 Asian. In the North, 27 youth participated: 14 females and 13 males; 16 participants identified as Indigenous (encompassing youth who self-identified as “Aboriginal”, “First Nations”, and “Indian”), 4 “White”/European, 4 multi-racial, 2 Métis, and 1 did not provide details.

### Data analysis

All interviews were transcribed and a thematic analysis was carried out to identify emergent themes. RADAR team members collaboratively generated 15 broad codes that captured youth experiences and reflections on mental health, substance use, and resilience. In this article, we primarily draw from the *Substance Use* code; as codes were not mutually exclusive, the *Substance Use* code also captures participants’ reflections on the intersections of substance use with the themes represented across the other 14 codes such as *Social Connections* and *Trauma and Stressors*. *Substance Use* included all participant references to alcohol, tobacco, cannabis, and other drug use as related to participants’ own experiences and those of friends and family. This code also captured participants’ reflections on substance use in their schools and communities more broadly.

The multi-site qualitative analysis undertaken for this paper builds on Herriott and Firestone’s analytical approach to case study data that spans more than one setting [41]. The authors argue that this approach can enhance the rigour of a study, while preserving depth and contextualization of research findings. In application of this approach to the qualitative data in this study, we seek to extend the application of multi-site qualitative analysis to ethnographic interview data from three geographical sites. Following thematic coding of all study data, analysis for this paper began with a within-site thematic coding and analysis of the *Substance Use* code for each of the three research sites [42]. Distinct analysis of data from each study community allowed for the preservation of the shared participant experiences and narratives within each site and ensured in-depth description of the ways in which substance use was uniquely situated [41]. Subsequently, a between-site analysis was conducted in which emergent themes were compared to uncover patterns across sites; through this analysis, the research team identified two broad themes at the level of the entire study population: youth context and experiences of use, and substance use management. To avoid “context-stripping”, in which research findings are removed from their local contexts, a second within-site analysis was conducted following the identification of themes to ensure that findings remained locally and contextually grounded [43].

## Results

Findings from our multi-site qualitative analysis illustrate the ways in which youth in each of the three sites took up informal harm minimization strategies based on the context of substance use in their peer groups and communities. The findings are reported separately for each of the three research sites in order to highlight location-specific themes. For each site, we provide contextual descriptions of the community and social settings in which youth substance use was situated, as well as participants’ experiences of substance use. We then present the strategies that youth employed to manage and minimize the harms of their own substance use.

### The City

#### Context and experiences of use: parties and popularity

Youth participants in The City described a peer and school context in which substance use was not the norm. Many participants stated that they did not drink alcohol or use drugs and those who did engage in personal use described occasional or infrequent use of alcohol or cannabis. For example, one participant describing cannabis use stated: “it’s like drinking, you do it once every three months, not at the same time of course.” When participants did consume alcohol, it was almost exclusively in the context of parties. In describing a typical party, many participants characterized the event solely through substance use: “a lot of drinking”, “get piss assed drunk and do stupid things”. For many youth, partying was synonymous with substance use; for example, one participant referenced friends who “party a lot…by party I mean like, they drink.” The connection between parties and drinking was understood as not only a school norm, but also a cultural norm: as one participant reflected, “it seems like a typical high school experience.”

Of all the substances discussed by participants, what the participants referred to as “hard drugs”—including drugs such as cocaine and methamphetamine—were used the least in their school and peer group. Many interviews suggested this form of substance use was outside of shared social expectations in the youth community. As one participant stated:

There’s always a rumour that someone snorted a line of cocaine or something and you’re just like “someone did that?” Like, “sorry this isn’t like the Bronx or something. We usually don’t do that here.” That kind of stuff, that’s really a shock…

Participants often described individuals who did use hard drugs as “popular” or “cool”—one participant speculated that a friend who frequently talked about drug use was simply “trying to be all cool and everything”, though did not believe that this friend actually used substances. Yet despite the social cachet of substance use, individuals who did use drugs were frequently described by participants in negative terms. Substance use was viewed as an indicator of low intelligence—“the people who do that generally aren’t the smartest”—or connected with adverse outcomes in adulthood such as homelessness and mental illness—“crazy people” or “hobos”. One participant described an incident in which a classmate was bullied online regarding her “drug addictions”, demonstrating that despite associations of drugs with a popular crowd, the use of hard drugs was not socially endorsed.

In contrast, cannabis use was reported by many, and frequently described in positive terms: “quite nice”, “just makes you happy”. Participants regularly invoked the neighbourhood, city, and provincial context as explanatory for its ubiquity: “weed is everywhere, this is [name of neighbourhood]”, “this is [The City], lots of kids smoke weed”, “it’s BC, it’s peaceful, everyone smokes weed.” Many participants explained that peers were often relatively open about their use—the prevalence of cannabis use was known throughout the school, and peers were described as smoking across the street from the school (in order to be technically off of school grounds) in plain view. In the context of commonplace cannabis use, many youth characterized substance use education as equating the harms of different drugs including cannabis, which was not reflective of youth perspectives. This lack of clarity in messaging led one young man to reflect:

They make weed sound so bad, and then when people find out that weed’s not that bad, they think all drugs are not that bad, so then they go into the drug thing, and they think that all drugs won’t change them, but those other drugs change you.

For youth, exaggerating the effects of some substances resulted in the minimizing of harms of others, and participants in The City called for honesty in describing the relative harms of substance use.

#### Substance use management: staying away and limiting use

Youth in The City predominantly maintained their own substance use limits through the strategy of staying away from contexts in which these limits may be exceeded by those around them. This involved both physical and social positioning to avoid particular events or peer groups. Participants described choosing to attend certain parties, while avoiding other “more intense ones” at which drugs may be present or alcohol use may be increased. One participant stated that young people in her peer group who she termed “the average people”

will have their own couple parties, but there’ll be alcohol but there won’t be any drugs. So that one’s fine, I’ll go to that one—but the other ones, you’ve got the popular people from different schools all going, and then you get a lot of those sort of people together and just generally isn’t a good idea.

Many participants who drank alcohol in moderation described attending and enjoying parties in which alcohol was present, but stayed away from contexts in which peers may “drink a lot and go overboard.” In addition to avoiding spaces of alcohol and drug use perceived as excessive may occur, participants also described staying away from individuals and social groups who used certain substances or had a particular pattern of use. One participant described that after a former best friend “turned to cocaine”, she felt that she “was not part of that crowd whatsoever, so [she] just left them all.” Staying away was articulated as a strategy for avoiding particular substances or patterns of substance use, but also for forming an identity as an individual who uses within the socially acceptable limits—as one participant stated, “I’ve noticed the people that do that, and the people that don’t do that…and who do I wanna be?” Staying away was viewed by youth as a form of self-protection from perceived harms, including getting “caught” by parents or caregiver, or experiencing negative effects, which were often described in non-specific terms: “like a zombie”, “you get messed”, “changes your brain”. Additionally, staying away also served to reinforce belonging in groups that share use patterns and perspectives on substance use—very few participants described having friends or social groups who used differently from themselves, and those who did described the isolating impact of this difference. One participant whose friends used but did not use himself stated “there’s so many things I don’t know about them”; conversely, one participant who smoked cannabis and described coming to school “baked” stated “people look—they treat you different of course.” For youth in The City, a predominant strategy for managing substance use was staying away from particular contexts—both parties and peer groups—that young people perceived as having potential for substance-related harms.

When participants in The City chose to use substances, most utilized the strategy of limiting their own use: using in moderation (in frequency or amount) and intentionally selecting the types of substance used. Many participants described the approach of moderation to drinking or smoking cannabis, for example limiting alcohol consumption to “once every like three months or once every six months, which amounts to pretty much never.” Another participant described himself as “not really that much of a drinker” and stated that at parties he “might have a beer or something.” Youth also avoided particular substances while continuing to use others—frequently, this involved using alcohol and cannabis while avoiding “hard drugs”. One participant shared:

I personally smoke weed sometimes on the weekends, I’m not going to lie, I’ve never done other drugs…Then there’s also like the kids doing coke and all this stuff but I’m not into that, my friends are not really into that. I know some people that do this stuff but it’s not my thing, it’s not my life, I don’t really care. There’s this drug that people do, I don’t really know these people, but it’s like meth, and it’s like in five years you’re gone, I mean it’s messed up.

As in this example, many participants cited concerns about potential harms as a primary reason for not using particular substances. Participants did not explicitly draw on formalized sources of drug education, such as at school or in the community, in informing these choices. Rather, knowledge of these harms was often drawn from witnessing individuals in the community who the participants interpreted as being negatively impacted by drug use. For example, one participant who smoked “a lot of weed” but chose not to use other drugs described seeing individuals in a particular neighbourhood in The City:

…whenever I see those people…like one girl’s just standing there and she’s hitting herself in the head or something, like saying “get out of my head, get out of my head” smashing—it’s like “oh my God! Don’t do drugs.” It’s like a warning: “Don’t do drugs. You end up messed up.”

### The Valley

#### Context and experiences of use: social divisions and peer influence

Youth in The Valley described their social context as stratified on the basis of substance use. Despite many participants reporting substance use as widespread in the school, many others stated that they “don’t really know anybody who uses” or described themselves as “sheltered”. Interaction between those who used substances and those who did not was limited: “It’s mostly *those* guys dating *those* girls that are into that kind of stuff I guess.” Participants described “rivalries” and “tensions” between the two groups, and one youth stated, “most of us just kinda wanna get through high school and never wanna see some of these kids again… ‘cause we’re going to be going on to university while they’re still here doing drugs.” As in this example, participants who did not use substances tended to hold strong negative views of peers who did use, characterizing substance use as a “stupid decision”, and individuals who use as “bad” or “dangerous”. One participant in describing the two “completely separate” groups of the school’s social scene differentiated between those who are “all clean, they’re all good, they don’t do drugs” and “the ‘bad’ people…the bad part of school”. Participants who formerly used substances but had since stopped drew on similar descriptors in discussing their own previous use. One participant explained, “I was going down a really rough road” before becoming “all clean”; another participant commented “I did a lot of stupid things, and boy…I’m glad that I’m out of it.” Many participants associated substance use with violence, describing individuals who use substances as “always starting fights” and carrying weapons. Youth who previously used substances also endorsed this association of alcohol and drug use with other behaviours such as bullying or stealing cars, however contextualized their history of use as a coping strategy for managing “extreme stress” and other difficult emotions. Some individuals who did not use substances suggested that drug use may be a “bad way to cope” but many struggled to make sense of their peers’ substance use: “…other kids are blatantly bad like drugs and alcohol and that kind of thing, right? *Fights* for no reason.”

Though participants described distinct social groups and limited interaction between youth who used substances and those who did not, the danger of being influenced into using was viewed as a present and real concern. Individuals who used substances were frequently described as “bad influences” by those who did and did not use alike. However, substance use, described almost exclusively in negative terms, was not itself viewed as appealing or tempting. Rather, participants suggested that proximity to individuals who used substances was a risk factor for being influenced to use:

There are some students out there, probably aren’t the best of friends they could be…So we just feel safe being in that group away from friendships that could be dangerous…Like it could lead to things in a friendship, that maybe you wouldn’t’ve gotten yourself into? Things like, I guess, like smoking and drugs and things like that…There could be some people that could cause you to go down a road like that.

While one participant suggested that individuals who use drugs “recruit”—“they might meet the little kid at the gym…and slowly get them to smoke marijuana”—most participants described this potential for negative influence in non-specific terms. One young man described his own introduction to substances use as “I guess I just met more people and started smoking weed with these people”, while another participant who did not use speculated that others are influences: “maybe someone, a relative or some friend, who is just like ‘oh yeah, he’s doing that, should I do that now?’” Many youth expressed concern regarding the potential for being influenced to use substances, and fear of this influence reinforced social divisions between social groups.

#### Substance use management: social avoidance and positive influence

In accordance with the common perception of substance use as harmful and “dangerous”, many participants in The Valley made efforts to minimize potential harms by avoiding individuals and social groups who used. Many participants described evading friendships with people who used substances, and one individual stated that he kept his distance from “these kids” in the hallways. Avoiding individuals who used substances and fostering relationships with peers who did not use gave many participants a sense of belonging: “we have similar values…so that’s really what’s drawn us together as friends.” One participant described breaking up with a partner due to his substance use, while another participant shared that she would avoid entering into romantic relationships with someone who might “pull me down to a lower level.” Participants consistently viewed friendships with peers who did not use as protective from the perceived harms involving substance use, and avoided interaction with individuals characterized as “bad influences”.

When youth did engage with peers or friends who used substances, they often attempted to exert what they viewed as positive influence. Among participants who both used and did not use themselves, they described encouraging friends to stop or reduce their use. One participant shared: “In grade 10, my friend was going to start dealing, and me and my friend just took him into a corner and beat the crap out of him. We’re like ‘what are you doing?’ So he didn’t deal.” Another participant stated that even in the context of his own substance use, “I try to be the best influence I can…I try to be a big brother sort of person to other people, making sure they’re making the right choices.” These “right choices” included not using at school, and using only certain drugs. Attempts to influence friends or partners’ substance use were not always successful: participants who used substances described lying to partners about their use or experiencing relationship difficulties. One individual who smoked cannabis stated that her boyfriend “wants me to respect the fact that he doesn’t like it, so I shouldn’t do it either” but had not stopped her use. In addition to youth reflections on the influences of peers, some participants viewed teachers as having the ability to positively influence youth. One participant stated “if they’re just comfortable talking about it normally with students, they’ll feel that they can actually ask questions about it, right?” However, youth described teachers as not consistently confident in discussing substance use with students.

### The North

#### Context and experiences of use: boredom and availability

Substance use in The North was described by participants as commonplace among their peers, and most participants in the study used alcohol or drugs. Youth described the predominant influence for substance use as boredom. The North was described as having few services, activities, or spaces for young people, and most participants repeated the refrain of “there’s nothing really to do”. In the context of this boredom and lack of opportunities, participants regarded substance use as an activity itself—one participant stated, of cannabis, “it just gives me something to do”. Many participants echoed this notion, listing alcohol and cannabis use as the primary activities for “kids our age”. For example, one participant stated, “All there really is to do in this town is drink, smoke pot, and get into trouble.” Some participants were able to identify alternate activities, such as sports, as providing a relief from boredom and “help[ing] keep away the alcohol”, however, explained that these opportunities remain limited, and only beneficial “if you are athletic”. Youth described engaging in substance use as an activity to do on one’s own, with small groups of friends, and at parties. One young woman described a typical Friday night as “hanging out with friends, smoking up, and drinking…we find ways to entertain ourselves”, while a young man described his primary activities as watching TV or “once in a while I just go walk around, whenever I drink”.

The participants also described the environment of The North, as well as its lack of available activities, as a factor in the context of youth substance use. Youth described substance use as visible and prevalent in their community: “You go into town on Main Street, and you see people sitting there, bottles and bottles of alcohol and drugs.” While some participants described finding it easier to avoid substance use on the First Nations reserve, as the area’s only liquor store was located in town, others noted “a lot of drug selling and drinking that goes on on Rez”. Participants described relatively easy access to alcohol in both locations from “whoever’s willing to boot [provide alcohol to minors]”. One participant’s narrative addressed the inter-generational impacts of alcohol availability, as adults who “have already went downhill” in turn sold to young people: “The alcoholics in town, they don’t care—like, ‘if you’re going to support for my next bottle, then hey, I’ll get you yours’”. In the context of inter-generational use, many participants grappled with the narrative of substance use as inevitable in The North. While some participants actively rejected this narrative for themselves, others spoke to the distress of identifying their own use as consistent with this societal narrative. As one 17-year-old First Nations participant stated: “I wish I didn’t skip—flunk out on school and, and just become one of those reserve Indians that just party around, smoke weed and whatnot.”

#### Substance use management: using selectively and reducing use

In The North, harm minimization strategies predominantly arose from witnessing and/or experiencing negative effects of substance use. Some participants described personal experiences of substance use that contributed to their desire to use differently. For example, one participant shared: “I went on a complete binge, never going to do that again. I can see why a lot of people are messed up from drinking so hard core, really harsh on your body.” For other participants, witnessing negative effects of substance use in their families and communities shaped their perspectives on their own use. One participant described negative experiences of a family member’s drug use: “it ended up making her hit my mom and steal money and all that from us…And it’s just like, I don’t want to do that…What’s the point if it’s going to mess you up that bad?” Stemming from witnessing harmful aspects of substance use, many participants managed their use through using selectively—choosing to use particular substances and avoiding others. A common substance of choice was cannabis, which was described as a “natural herb” and “the main thing that wouldn’t make you crazy”, while other drugs were frequently avoided. One young man gave his rationale for not using drugs as “my family members are into hard drugs and stuff, so I’ve seen that they fuck shit up.” Other participants described certain drugs as “more harsh” or “gross”, and some discussed the concern of substance use challenges: “It is fun for the first time or two, then you get addicted or something and then you just need it, need, need, need. I don’t want to be addicted.” Participants who used cannabis often spoke adamantly about not using other drugs nor wanting to use them: “never touched it [cocaine]”, “really don’t wanna do it [other drugs]”, “do not wanna try anything else”, “absolutely not”.

In addition to using selectively to avoid perceived harms of particular substances, many participants also sought to minimize harms by reducing use. For many of the participants, the desire to reduce their use followed negative experiences: some described a single incident, such as blacking out when drinking and subsequently losing items or incurring injuries. Others described an accumulation of negative effects—one participant explains reducing her alcohol intake because “I don’t think my liver could really take the hard stuff anymore…The doctors are saying that I’ve been drinking too much hard stuff…there could be something wrong with my stomach.” As well as managing negative effects of substance use, many participants reduced their use in order to focus on other goals including attending school. Multiple participants had received school suspensions for drinking or smoking at school or attending school intoxicated, and articulated clear goals to “keep in school this year”. One participant described smoking cannabis “lots last year, but…I actually didn’t go to school lots last year, so this year I just [need] to buckle down, actually to go to school.” Another participant reported cutting down on cannabis and alcohol consumption during the school year, “but during the summer, drink a little too much.” A few individuals viewed reducing or abstaining from use as a strategy for achieving the level of success required to attend university or get a good job, and limited their own use with these goals in mind.

Though many participants reflected on the harms of substance use and demonstrated their use of individual strategies to minimize these harms, youth also acknowledged their perceptions of positive effects of substance use. Some of the youth described using drugs as “a good trip” or having the effect of “mak[ing] me excel at stuff”. Others described using substances to manage difficult emotions, including to control anger, “keep me calm”, or simply to “get through the day”. In this context in which youth articulated perceived benefits from substance use, and in which use was commonplace, abstinence-based messaging was described as unrealistic and unhelpful. One young woman commented “they have a lot of school awareness things but it seems like a lot of the people like know that already, and they still just do it, so I think maybe, instead of telling kids not to do it at all, they should tell them how to do it safer at least.”

## Discussion

Our findings illustrate that youth experiences, perspectives, and strategies related to substance use are deeply situated within geographical, social, and cultural contexts. In The City, cannabis is openly discussed and frequently used by youth, alcohol is a foundational element of a “typical” party, and “hard drugs” are viewed as dangerous. In The Valley, type and frequency of use divides social groups, with youth who avoid alcohol and drugs characterizing substance use as harmful and against common sense. In The North, alcohol and cannabis use is commonplace, yet many of the youth discuss reducing use and avoiding particular substances due to witnessing the potential for harmful effects within their families and community. Youth, regardless of their own pattern of use, describe their substance use experiences and decisions in relation to the broader social context of their friends, peer groups, and community; youth variously describe their use as congruent with, or explicitly in contrast to, the dominant contextual experience in their communities.

The emergence of a shared narrative of substance use across youth interviews within each research site is consistent with the concept of “differentiated normalization”, which emerged from a critical analysis of Measham, Newcombe, and Parker’s [44] theory of normalization. While normalization suggests that drug use has shifted within youth culture from the fringe to the mainstream, differentiated normalization posits that individual substances and modalities of use become variously normalized within different geographic and social contexts [45]. Differentiated normalization has been utilized within the youth substance use literature to frame the geographic differentiation of youth urban/rural substance use trends [46] and to understand the impact of socio-political contexts within which particular substances, such as cannabis, become normalized within youth populations [47]. In our study, youth descriptions of the context for substance use reveal both the differentiated normalization of substance use behaviours and perspectives within each community context, and demonstrate youth’s awareness of the ways in which their individual experiences of substance use are shaped by particular geographic, socio-cultural, and political factors—for example, ways in which governmental policy and legislation is taken up in youth attitudes toward cannabis use in The City, or the ways in which a small town identity in The North informs youth characterization of substance use as an activity to counter boredom.

In addition to local contexts shaping the normalization of particular substance use trends, youth substance use perspectives and experiences in this study were also informed by the neoliberal conceptualization of individual responsibility for substance use and associated harms [23]. In The City and The Valley, substance use was framed as a poor decision by both users and non-users, mirroring the “individual deficit” perspective frequently taken up in substance use education and public health messaging [19]. While peer influence was identified as a contextual factor for use in The Valley, youth-centred individual responsibility and decision-making, framing use and abstinence as personal choices as opposed to situationally and structurally mediated states [48]. Further, in The North in particular, historical and ongoing colonialism impacted youth perspectives on their own use and the context of use in their community. O’Gorman [49] argues that normalization of substance use not only occurs within geographic and socio-cultural contexts but also results from the intersectional context of class, race, and gender. It is through these complexities that substance use can be simultaneously normalized and positioned as deviant [50]. In The North, one participant’s expression of distress at “becom[ing] one of those reserve Indians” highlights the ways in which pervasive colonial narratives of substance use in First Nations communities have positioned contextually normalized patterns and types of substance use as deviant. In each of the three communities, youth experiences of substance use were contextualized within particular social and community norms, but were also informed by neoliberal and colonial framing of responsibility and blame for particular contexts of use.

Viewing youth substance use through a critical lens of differentiated normalization is crucial not only for framing the context of use for youth within the three communities in this study but also for understanding youth’s strategies for minimizing harms. Youth in The City speak to selecting social events that were likely to involve use that was consistent with their own use patterns and preferences, and discussed limiting their frequency and type of substance use in accordance with their personal beliefs surrounding moderation. In The Valley, many participants described forming friendships that were perceived as protective from substance use harms; youth who did use frequently attempted to influence the substance use behaviours of others in what they viewed as a positive direction. Youth in The North also described selectively using particular substances as a harm minimization strategy for their own use; additionally, substance use reduction and moderation were frequently cited, with youth maintaining and shifting their personal limits based on their experiences and aspirations. In each of these settings, youth developed and maintained harm minimization approaches to their own substance use that allowed for the navigation of the contexts of substance use in accordance with their own beliefs and preferences. Many of the youth we interviewed described the perceived positive effects of substance use, including pleasure and enjoyment, and managing difficult emotions—yet also described the ways in which they engaged in “bounded consumption” supported by harm minimization strategies that limited their pleasure-seeking and balanced their use with other responsibilities and desires [24, 51].

## Conclusions

This study suggests that youth are actively engaging in strategies to minimize the harms of substance use within their local contexts—yet are doing so on an informal or ad hoc basis. The development and implementation of substance use programming that is informed by evidence from harm reduction approaches is needed in order to resonate with young people. Further, for harm reduction programing to be effective, it must also be informed by youth experiences [21] and, as youth called for in this study, honesty. Multi-site analysis of findings across research sites in this study has illustrated the differences in the ways in which substance use is taken up by youth based on a range of individual and community factors. While some youth substance use approaches have sought cultural relevancy in programming, for example, for First Nations communities [52], content must also hold specific relevancy to young people’s “actual attitudinal and behavioural norms”, including types of substances used, patterns of use, and informal harm minimization strategies utilized by youth [53]. Harm reduction within a framework of culturally, socially, and geographically differentiated normalization, as supported by the findings from this study, holds the potential to speak directly to the ways in which youth use and manage their use and thereby support youth resilience and may reduce the possibility of harms associated with substance use.
